# Assessment of the contribution of *APOE* gene variants to metabolic phenotypes associated with familial longevity at middle age

**DOI:** 10.18632/aging.101017

**Published:** 2016-08-18

**Authors:** Raymond Noordam, Charlotte H. Oudt, Joris Deelen, P. Eline Slagboom, Marian Beekman, Diana van Heemst

**Affiliations:** ^1^ Department of Internal Medicine, Section of Gerontology and Geriatrics, Leiden University Medical Center, Leiden, the Netherlands; ^2^ Section of Molecular Epidemiology, Department of Medical Statistics and Bioinformatics, Leiden University Medical Center, Leiden, the Netherlands

**Keywords:** human longevity, APOE, mediation, glucose, 25-hydroxyvitamin D

## Abstract

Offspring of long-lived families are characterized by beneficial metabolic phenotypes in glucose and lipid metabolism and low 25-hydroxyvitamin D. Although the genetic basis for human longevity remains largely unclear, the contribution of variation at the *APOE* locus has been repeatedly demonstrated. We aimed to assess whether ApoE isoforms mark the familial longevity status in middle age and subsequently to test to what extend this association is mediated by the metabolic characteristics marking this status. From the Leiden Longevity Study (LLS), we included offspring from nonagenarian siblings and partners as controls. Using the metabolic phenotypes of familial longevity as mediators, we investigated how *APOE* gene variants associated with LLS offspring/control status (in 1,515 LLS offspring and 715 controls). Within the LLS (mean age = 59.2 years), ApoE ε4 was not associated with a lower likelihood of being an LLS offspring, whereas ApoE ɛ2 was significantly associated with a higher likelihood of being an LLS offspring (odds ratio = 1.43), but this difference was not mediated (p-values>0.05) by any of the investigated metabolic phenotypes (e.g., diabetes and glucose). Therefore, variation at the *APOE* locus may not influence familial longevity status in middle age significantly through any of the metabolic mechanisms investigated.

## INTRODUCTION

Improved housing conditions and medical care are important causes of the increase in overall life expectancy over the past decades [1], but human life span is also partly genetically determined [2]. However, although various candidate genes have been identified that promote human longevity, the only gene identified by genome-wide association studies to be robustly associated with human longevity is *APOE* [3, 4], which encodes for the 299 amino acids long ApoE protein. Different isoforms for the ApoE protein (notably ε2, ε3 and ε4), coded by 2 SNPs (notably rs7412 and rs429358) in the coding regions of the *APOE* gene, have been studied in relation to multiple clinical outcomes [5, 6]. For example, the ε4 isoform has been associated with an increased risk of Alzheimer's disease, dementia, and coronary heart disease (CHD) and a higher serum level of low-density lipoprotein (LDL) cholesterol, compared to the ε3 isoform [7, 8]. The ε2 isoform has been associated with a lower risk of CHD and a lower LDL cholesterol serum level [8].

**Figure 1:**
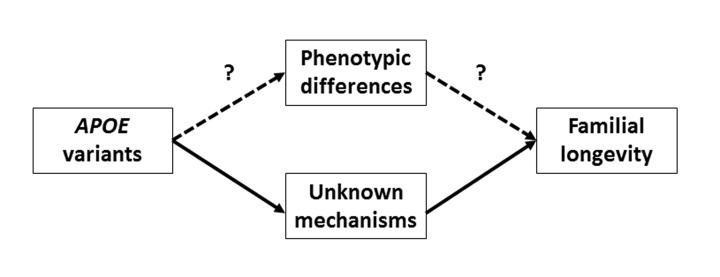
Graphical presentation of the biological routes through which *APOE* could affect familial longevity “Intermediate phenotypes” denotes the metabolic differences between offspring and controls in phenotype (e.g., lower level of serum glucose, lower level of serum 25-hydroxyvitamin D, lower frequency of type 2 diabetes, in LLS offspring compared with controls). In this study, we aim to assess whether this route is plausible.

The Leiden Longevity Study (LLS) has been designed to investigate the genetic basis and phenotypic characteristics of human familial longevity based on the inclusion of nonagenarian siblings and their offspring [9]. In the nonagenarian LLS siblings, lower IGF-1/IGFBP3 levels were associated with increased old age survival [10] and gene set analysis of GWAS data revealed significant differences in the insulin/insulin-like growth factor (IGF-1) signaling (IIS) pathway when LLS nonagenarian sibling were compared to younger controls [11]. Importantly, the offspring from nonagenarian siblings (denoted hereafter as LLS offspring) had, compared to age/matched controls, a lower prevalence of type 2 diabetes and myocardial infarction [12]. In addition, in participants without diabetes, LLS offspring had a favourable glucose-insulin homeostasis [13-15], and in the total study population, LLS offspring had a beneficial lipid profile [16], lower thyroid function [17-19], altered mTOR signalling [20], and a lower serum level of 25-hydroxyvitamin D [21]. Despite these metabolic characteristics and the evidence of genetic enrichment for extended lifespan in these families [9], genetic variation generally associated with diabetes or 25-hydroxyvitamin D, did not explain the difference in glucose-insulin homeostasis or the serum level of 25-hydroxyvitamin D between the LLS offspring and controls [21-23]. A composite score of genetic variants (including a variant in *APOE*) associated with high serum LDL cholesterol level was, however, significantly lower in offspring compared with controls [24] Here we intend to investigate whether longevity associated variants at *APOE* mark familial longevity status (LLS offspring/control status) in middle age and whether this is mediated by the beneficial metabolic phenotypes typical for longevity families, as highlighted in Figure 1. Results from such analyses will contribute to a better understanding of whether variation in *APOE* contribute to the metabolic characteristics of human longevity in middle age.

## RESULTS

### Characteristics of the study population

A total of 2,230 participants (1,515 LLS offspring and 715 controls) were successfully genotyped and had data available on non-fasted serum measures. Table 1 presents the characteristics of the whole study population as well as stratified for offspring and controls. Of the tested phenotypes, serum glucose, insulin, triglycerides, and 25-hydroxyvitamin D were significantly lower, while serum HDL cholesterol was significantly higher in the LLS offspring as compared to controls. Furthermore, the LLS offspring had a significantly lower prevalence of diabetes, hypertension and MI than controls.

**Table 1:** Study variables compared between offspring and controls

	All (N = 2,230)	Offspring (N = 1,515)	Controls (N = 715)	P-Value
**Demographics**				
Age in years	59.2 ± 6.8	59.3 ± 6.5	58.8 ± 7.4	0.138
Female, n (%)	1,212 (54.3)	803 (53.0)	409 (57.2)	0.092
Body Mass Index in kg/m^2^	25.4 ± 3.5	25.3 ± 3.5	25.6 ± 3.6	0.066
**Glucose-insulin homeostasis**^a^				
Glucose in mmol/L	5.8 ± 1.1	5.7 ± 1.1	5.9 ± 1.2	**0.002**
Insulin in mU/L, median (IQR)	16.0 (9.0-28.0)	16.0 (9.0-28.0)	17.0 (9.0-29.0)	**0.034**
**Serum lipid levels**^b^				
Triglyceride in mmol/L, median (IQR	1.5 (1.1-2.2)	1.5 (1.1-2.2)	1.6 (1.1-2.3)	**0.003**
Total cholesterol in mmol/L	5.6 ± 1.2	5.6 ± 1.2	5.7 ± 1.1	0.263
HDL cholesterol in mmol/L	1.5 ± 0.5	1.5 ± 0.4	1.4 ± 0.5	**0.040**
LDL cholesterol in mmol/L	3.4 ± 0.9	3.4 ± 1.0	3.4 ± 0.9	0.359
**Other serum levels**				
25-hydroxyvitamin D in nmol/L, median (IQR)	65.6 (52.7-80.6)	64.4 (51.5-79.6)	68.3 (55.5-83.3)	**<0.001**
**Disease history**				
Diabetes Mellitus, n (%)	113 (5.1)	61 (4.0)	52 (7.3)	**0.002**
Hypertension, n (%)	585 (26.2)	357 (23.6)	228 (31.9)	**<0.001**
Myocardial infarction, n (%)	56 (2.5)	31 (2.0)	25 (3.5)	**0.039**

Abbreviations: n, number of participants; IQR, interquartile range; SD, Standard deviation. Data presented as the mean ± SD unless indicated otherwise.

a Participants with diabetes were excluded.

b Participants using lipid lowering agents were excluded.

Missing values: Body Mass Index, 315; glucose, 13; insulin, 61; HDL cholesterol, 1; LDL cholesterol, 63. Comparisons between offspring and partners were adjusted for age and sex, and corrected for familial relationships using clustered robust.

**Figure 2:**
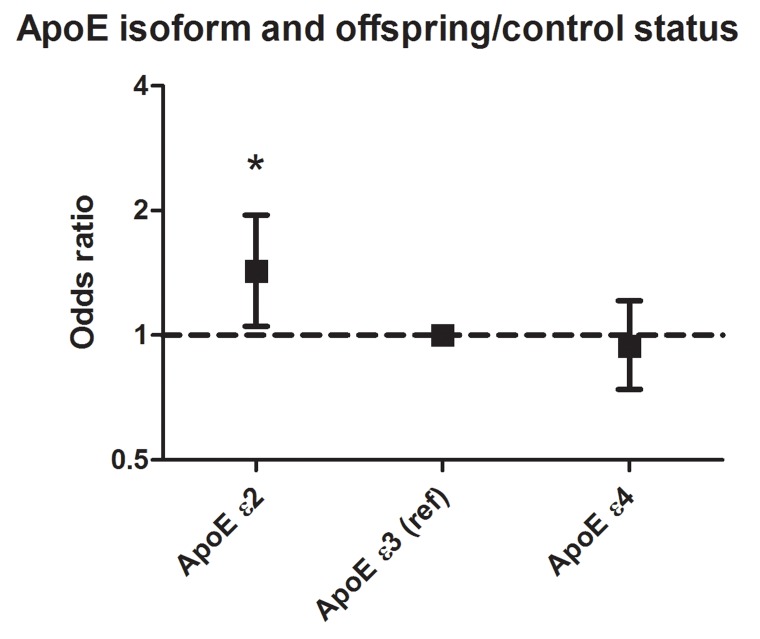
Association between ApoE isoforms and propensity to be an LLS offspring Carriers of the ApoE ε3 isoform were used as reference. Analyses were adjusted for age and sex, and corrected for familial relationships using robust standard errors. A total of 300 participants was ApoE ɛ2 carrier, 1369 participants carried ApoE ɛ3/ɛ3, and 499 participants carried ApoE ɛ4. For these analyses, 62 participants carrying the ε2/ε4 isoform were excluded.

After adjustment for age, gender, and correction for familial relationship, ApoE ε2 carriers had a higher likelihood of being a LLS offspring than a control (OR [95%CI]: 1.43 [1.05 – 1.95]; Figure 2). However, carriers of the ApoE ε4 isoform had a similar likelihood of being either an LLS offspring or control (OR [95%CI]: 0.94 [0.74 – 1.21]). Similarly, the likelihood of being an offspring increased significantly per additional copy of the rs7412-T (ApoE ε2) allele (1 copy: odds ratio [95% confidence interval] = 1.34 [1.01 – 1.78]; 2 copies: odds ratio [95% confidence interval] = 1.76 [0.58 – 5.35]; p-value_trend_ = 0.03). No significant association was observed with rs429358 (p-value_trend_ = 0.19).

### APOE and phenotypes of familial longevity

The associations between variation in *APOE* and metabolic phenotypes of familial longevity are presented in Table 2. Per additional copy of the ApoE isoform (coded as: ε2 carrier = 0, ε3/ε3 = 1, ε4 carrier = 2), HDL cholesterol serum level decreased (p-value_trend_ = 0.02) as well as the risk of diabetes (p-value_trend_ = 0.04). No significant differences were observed for the metabolic phenotypes when ApoE ε2 and ApoE ε4 carriers were separately compared with ApoE ε3. For the individual SNPs (Table Supplementary 1), an additional copy of the rs7412-T allele was associated with a higher risk of diabetes (β_additive_ = 0.40; p-value_trend_ = 0.04), and the mean level of serum triglycerides and HDL cholesterol were higher, although marginally not statistically significant (p-values_trend_ < 0.10). Further-more, per additional copy of the rs429358-C allele, the level of HDL cholesterol in serum was nearly significantly lower (p-value_trend_ < 0.10)

**Table 2:** Association between genetic variation ApoE isoforms and phenotypes of familial longevity at middle age

	ApoE ε2 carrier		ApoE ε3 (ref)	ApoE ε4 carrier		P-value_additive_
	β (SE)	P-Value		β (SE)	P-Value	
**Glucose-insulin homeostasis^a^**						
Glucose in mmol/L	0.06 (0.08)	0.47	0 (ref)	0.00 (0.06)	0.95	0.51
Insulin in mU/L^c^	0.01 (0.05)	0.84	0 (ref)	−0.04 (0.04)	0.37	0.31
**Serum lipid levels^b^**						
Triglycerides in mmol/L^c^	0.07 (0.04)	0.08	0 (ref)	0.05 (0.03)	0.09	0.94
HDL cholesterol in mmol/L	0.04 (0.03)	0.24	0 (ref)	−0.04 (0.02)	0.09	**0.02**
**Other serum levels**						
25-hydroxyvitamin D in nmol/L^c^	−0.03 (0.02)	0.15	0 (ref)	0.01 (0.02)	0.41	0.12
**Disease history**						
Diabetes, yes	0.21 (0.26)	0.42	0 (ref)	−0.42 (0.29)	0.14	**0.04**
Hypertension, yes	−0.02 (0.16)	0.88	0 (ref)	−0.16 (0.13)	0.23	0.27
Myocardial infarction, yes	0.43 (0.36)	0.23	0 (ref)	−0.17 (0.37)	0.64	0.17

a Participants with diabetes were excluded.

b Participants using lipid-lowering agents were excluded.

c Depicted beta is log transformed.

Analyses were adjusted for age, gender, and corrected for familial relationships using clustered robust standard error. P-value_additive_ depicts the statistical significance of the additive effect of the ApoE isoforms (ε carriers = 0, ε3/ε3 = 1, ε4 carriers = 2) on the outcome. A total of 300 participants was ApoE .2 carrier, 1369 participants carried ApoE .3/.3, and 499 participants carried ApoE .4. For these analyses, 62 participants carrying the ε/ε4 isoform were excluded. P-values <0.05 are presented in bold.

### Mediation by phenotypes of familial longevity

Table 3 presents the mediation estimates of the metabolic phenotypes of familial longevity for the previously observed association between ApoE ε2 and LLS offspring/control status. Overall, none of the metabolic phenotypes investigated significantly changed the effect size of the association between ApoE ε2 variation and LLS offspring/control status (p-values for all metabolic phenotypes investigated >0.05). For example, circulating insulin levels accounted for 2.6% of the association between ApoE isoforms (ApoE ɛ2 vs ApoE ɛ3/ɛ3) and LLS offspring/control status.

**Table 3:** Assessment of mediation of the association between ApoE ε2 and offspring/control status by phenotypic differences

	β basic model^a^	β basic model + mediator^a^	Mediation, %^b^	p-value^c^
**Glucose-insulin**				
**homeostasis^d^**				
Glucose	0.30	0.30	<0%	0.45
Insulin	0.30	0.27	2.6%	0.80
**Serum lipid levels^e^**				
Triglycerides	0.39	0.42	<0%	0.10
HDL cholesterol	0.39	0.38	1.1%	0.42
**Other serum measures**				
25-hydroxyvitamin D	0.36	0.34	1.7%	0.15
**Disease history**				
Diabetes	0.36	0.37	<0%	0.48
Hypertension	0.36	0.36	<0%	0.77
Myocardial infarction	0.36	0.37	<0%	0.41

a Beta estimate depicts the difference in outcome (log(odds) of the propensity to be an LLS offspring) between ApoE ε2 carriers and ApoE ε3 carriers (reference).

b Negative mediation estimates (e.g., the association becomes stronger after adjustment) are denoted as “<0%”.

c P-values determined using bootstrap method (1000 runs).

d Participants with a history of diabetes mellitus were excluded.

e Participants using lipid lowering medication were excluded.

A total of 300 participants was ApoE ɛ2 carrier, and 1369 participants carried ApoE ɛ3/ɛ3. Participants carrying the other *APOE* gene variants were excluded.

Similarly, when we compared the metabolic phenotypes of familial longevity in middle age between LLS offspring and controls in a subpopulation of individuals all carrying the ApoE ε3/ε3 isoform, the results were not substantially different as compared with the total study population (Table Supplementary 2). Also, when we calculated residuals additionally adjusted for ApoE is form for the serum measures, results remained similar (Table Supplementary 3).

## DISCUSSION

Within our study population, *APOE* genes variants, specifically ApoE ε2, were associated with the likelihood of being an LLS offspring. Of the investigated metabolic phenotypes, we found significant association between ApoE isoform and serum HDL cholesterol and diabetes risk (also rs7412). However, we found no evidence that the association between ApoE isoforms and LLS offspring/control status was mediated (e.g., reflected by low mediation estimates) by any of the metabolic phenotypes that were different between LLS offspring and controls. These results indicate that variation at the *APOE* locus may not influence familial longevity status in middle age through any of the metabolic mechanisms investigated.

The association between *APOE* and propensity of human longevity has been observed in multiple previous studies. In a genome-wide association study on surviving to ages above 90 years (in which also the nonagenarian siblings of the LLS participated), genetic variation in *APOE* and in a locus on chromosome 5q33.3 were the two loci identified to be different between middle-aged (<65 years) and long-lived (>90 years) individuals [3]. The identified variant at the *TOMM40*/*APOE/APOC1* locus, rs4420638, is in high linkage with ApoE ε4, and had a lower frequency in the long-lived individuals. Furthermore, other genetic association studies showed that the frequency of ApoE ε2 was slightly higher in nonagenarians compared with middle-aged individuals, but that the difference in frequency was largest for the ApoE ε4 [30-33]. A systematic review and meta-analysis, however, did not show a significant association between ApoE ε2 and exceptional longevity [34]. These findings are therefore in contrast with the findings of the present study in which we found that specifically ApoE ε2 was more common in the LLS offspring as compared to controls in middle age; the frequency of ApoE ε4 was only slightly, and not significantly, lower in the LLS offspring. Potential reasons for this discrepancy might be the difference in definition of longevity compared and the difference in age of the participants included in the studies. In our study, we studied the genetic basis of familial longevity within selected families at middle age, whereas the genetic association studies also include individuals of high age [30-33]. Nevertheless, more studies are required to elucidate this difference.

Besides the expected associations with serum lipid measures, we additionally observed that ApoE ε2 was associated with a higher risk of diabetes [35]. As our study was conducted in a Caucasian population, this discrepancy may be due to ethnic differences. Interestingly, we did not find evidence of an association between ApoE isoforms and glucose and insulin levels, even though we did find an association between ApoE ε2 and diabetes. Nonetheless, these results were similar as compared to a study conducted in Chinese [36]. It has been hypothesized that the association between ApoE ε2 and risk of T2D is mediated by dyslipidaemia, although only minimally [25]. In addition to diabetes, preliminary evidence in both *in vitro* and epidemio-logical settings suggest that ApoE ε4 is associated with a higher serum level of 25-hydroxyvitamin D [26]. However, within our study population, we were not able to replicate these findings, although a trend toward higher levels of 25-hydroxyvitamin D in carriers of ApoE ε4 was observed. Possibly, our sample size was too small to observe an association, or seasonal variation may have affected our results in the direction of the zero hypothesis. Nevertheless, for both diabetes and 25-hydroxyvitamin D more research is required to elucidate the biological pathway underlying their associations with genetic variation in *APOE*.

This study has a few limitations. First, all blood samples were non-fasted and taken at a random moment of the day. This could have resulted in a higher random error, which could be one of the reasons that variation in *APOE* was associated with diabetes, but not with serum glucose levels in participants without diabetes. Nevertheless, we expect that this has not resulted in systematic error but to deviation of the associations in the direction of the zero hypothesis. Second, a limitation of this study was that not every offspring might be enriched for longevity, since possibly not all inherited the favorable predisposition for longevity of their long-lived parent. This could have diluted potential differences between offspring and controls. Third, although our study comprised a relatively large number of participants and was sufficiently powered [29], the number of participants carrying ApoE ε2 (N = 300) and ε4 isoforms (N = 499) was limited. Fourth, our study contained a selected number of phenotypes. Possibly, more phenotypes are associated with genetic variation in *APOE*. For example, offspring of nonagenarian siblings also showed a decreased thyroid function compared to controls [17-19]. However, we did not find evidence in literature that variation in *APOE* affects thyroid function, which was therefore not included in the present study. And finally, results from the present study should be interpreted as hypothesis generating and requires replication in independent cohorts with a similar design, data collection and sample size as the Leiden Longevity Study [9].

In conclusion, although the likelihood of being an offspring was significantly dependent on ApoE isoform, especially on ApoE ε2, we did not find evidence that the previously observed metabolic phenotypes of familial longevity in middle age mediated this association. Therefore, this variant may contribute to healthy ageing by other (metabolic) pathways than the ones investigated in the present study

## METHODS

### Ethical statement

The Leiden Longevity Study was approved by the medical ethical committee of the Leiden University Medical Center. Written informed consent was obtained from all participants.

### Study population

The present study was conducted in the Leiden Longevity Study. This study aimed to investigate biomarkers and genetic variation associated with familial longevity. A more detailed description of the design and recruitment strategy of the Leiden Longevity Study has been published previously [9]. In short, a total of 421 long-lived families were recruited, without selection based on health condition or demographics. Families were included when at least two long-lived siblings were still alive and fulfilled the age criteria of being at least 89 years for men and 91 years for women. In total, 1671 offspring of these long-lived individuals were recruited and 744 partners thereof as controls who represented the general Dutch population.

The present study population only comprised the middle-aged participants (offspring and controls). We only included participants on which successful *APOE* genotype data and non-fasted serum screening parameters were available.

### Genotyping

DNA was isolated using standard techniques (QIAamp blood maxi kit, QIAGEN, (Venlo, the Netherlands)). For determining ApoE ε2/ε3/ε4 isoforms, two SNPs in the *APOE* gene were genotyped using two Taqman SNP genotyping assays with the following assayIDs: C_904973_10 (rs7412, APOE ε2) and C_3084793_20 (rs429358, APOE ε4) (APPLIED BIOSYSTEMS, Foster City, USA). The assay was run on a 7900HT (APPLIED BIOSYSTEMS) according to manufacturer's instructions and genotypes were called using the Sequence Detection Software version 2.2 (APPLIED BIOSYSTEMS). The genotype call rate for the *APOE* genotypes was 98.7% [16]. Individuals with the ε2/ε3 or ε2/ε2 variant were denoted as ApoE ε2 carriers, and individuals with the ε3/ε4 or ε4/ε4 variant were denoted as ApoE ε4 carriers.

### Serum measurements

Serum was collected non-fasted at the study center. For the present study the following serum levels were included in the analyses: glucose, insulin, total cholesterol, LDL cholesterol, high-density lipoprotein (HDL) cholesterol, triglycerides, and 25-hydro-xyvitamin D. These metabolic serum measures have previously been shown to be different between the offspring and controls [13-16, 21], and have been previously associated with *APOE* genotype [7, 8, 25, 26]. All serum levels were measured using fully automated equipment. The levels of glucose, total cholesterol, HDL cholesterol, and triglycerides were determined with the Modular P2 analyzer from Roche (Almere, the Netherlands). Insulin was measured with the Immulite 2500 from DPC (Los Angeles, CA, USA). An electrochemiluminescence immunoassay was used to determine the level of 25-hydroxyvitamin D using a Cobas e411 analyzer from Roche Diagnostics (Almere, the Netherlands). The level of LDL cholesterol in serum was estimated using the formula by Friedewald [27]. For this, individuals with a triglyceride level >4.5 mmol/L were excluded.

### Other variables

We additionally collected information on Body Mass Index (BMI), history of hypertension, myocardial infarction (MI), and diabetes, and use of glucose-lowering agents, lipid-lowering agents, and antihypertensive agents. BMI was calculated from self-reported height and weight (BMI=Body weight [in kg] / height^2^ [in meters]). History of disease was obtained from the participants' general practitioners (based on questionnaires). Participants were considered to have a history of diabetes when diagnosed with diabetes by a general practitioner, when taking medication, or when having a non-fasted glucose level above 11.0 mmol/L.

### Statistical analyses

Characteristics of the study population are presented as the mean (with standard deviation), median (with inter-quartile range; for continuous variables with a skewed distribution only), or number of participants (for dichotomous outcomes). In addition, these variables (e.g., serum glucose and LDL cholesterol concentration) are compared between the LLS offspring and controls using linear regression models.

The association between genetic variation in *APOE* and LLS offspring/control status was assessed using logistic regression models. Analyses were conducted for the ApoE isoforms (ε2 carriers = 0, ε3/ε3 = 1, ε4 carriers = 2) as well as for the separate *APOE* polymorphisms rs7412 (CC = 0, CT = 1, TT = 2) and rs429358 (TT = 0, CT = 1, CC = 2). The results from these analyses are reported as odds ratio (OR) with 95% confidence interval (95% CI), which can be interpreted as the increased/decreased likelihood of being an LLS offspring when carrying genetic variation in *APOE* compared with the control population. These analyses were adjusted for age, sex, and corrected for familial relationship using clustered robust standard errors

Study outcomes (e.g., serum glucose, serum LDL cholesterol or diabetes prevalence) that were significantly different between LLS offspring and controls were used to study whether these variables were dependent on the ApoE isoforms. For the analyses on the ApoE isoforms, carriers of the ε2/ε4 isoform were excluded (N = 62). These analyses were adjusted for age, sex, and corrected for familial relationship using clustered robust standard errors. For the analyses on glucose and insulin, we excluded all participants with diabetes (N = 113); for the analyses on serum lipid levels, we excluded participants using lipid-lowering agents (N = 173). Whenever necessary, variables were ln-transformed to obtain a normal distribution.

We subsequently investigated whether the association between *APOE* and LLS offspring/control status was mediated by any of the metabolic phenotypes that are different between LLS offspring and controls (e.g., glucose, 25-hydroxyvitamin D [14, 21]). A graphical interpretation of the rationale of this analysis is presented in Figure 1. For the mediation analyses, we used the widely applied method of Baron and Kenny [28], implemented for dichotomous outcomes (in the current study the LLS offspring/control status) in the binary_mediation program in STATA. Mediation estimates were calculated using the following formula: ([β of model without potential mediator – β of model with potential mediator] / [β of model without potential mediator]) *100. P-values indicating whether the mediator contributed significantly to the statistical model were obtained using bootstrap methodology in STATA (also implemented in the binary_mediation program). Our study sample was sufficiently powered (power > 0.8) to detect significant mediation with small effect sizes between determinant, mediators and outcomes (e.g., Fritz *et al*, Δ’ = 0.14 between APOE gene variants and LLS offspring/control status [29]). In addition, the comparison of phenotypes between LLS offspring and controls was repeated for the participants carrying the ε3/ε3 isoform. Furthermore, residuals (age, sex-adjusted, and additionally *APOE*-adjusted) of the serum measures were regressed against LLS offspring/control status using logistic regression analyses.

Statistical analyses were performed using STATA 12 (Stata Corporation, College Station, TX, USA). Two-sided p-values below 0.05 were considered statistically significant.

## SUPPLEMENTARY DATA TABLES


