# Supplementary material for: Assessment of the contribution of APOE gene variants to metabolic phenotypes associated with familial longevity at middle age
Source: Aging (Albany NY). 2016 Aug 18;8(8):1790–9. doi: 10.18632/aging.101017 (PMC5032696; doi:10.18632/aging.101017)
Supplement: Supplementary file 1 [file aging-08-1790-s001.pdf]

SUPPLEMENTARY DATA

Supplementary Table 1. Association between genetic variation in APOE and phenotypes of familial longevity at middle age

	rs7412		rs429358	
	**β (SE)**	**P-Value**	**β (SE)**	**P-Value**
**Glucose-insulin homeostasis[a]**				
Glucose in mmol/L	0.03 (0.06)	0.63	-0.01 (0.05)	0.91
Insulin in mU/L[c]	0.01 (0.04)	0.87	-0.04 (0.04)	0.27
**Serum lipid levels[b]**				
Triglycerides in mmol/L[c]	0.06 (0.03)	0.08	0.03 (0.03)	0.20
HDL cholesterol in mmol/L	0.05 (0.03)	0.08	-0.03 (0.02)	0.08
**Other serum levels**				
25-hydroxyvitamin D in nmol/L[c]	-0.02 (0.02)	0.28	0.02 (0.02)	0.21
**Disease history**				
Diabetes, yes	0.41 (0.21)	**0.04**	-0.23 (0.23)	0.33
Hypertension, yes	-0.01 (0.13)	0.97	-0.18 (0.11)	0.12
Myocardial infarction, yes	0.45 (0.27)	0.10	-0.25 (0.30)	0.40

[a]) Participants with diabetes were excluded. [b]) Participants using lipid-lowering agents were excluded. [c]) Depicted beta is log transformed. Analyses were adjusted for age, gender, and corrected for familial relationships using clustered robust. Beta estimates are depicted as the additive effect of the effect alleles of the polymorphisms on the outcome. P-values <0.05 are presented in bold.

Supplementary Table 2. Comparison offspring and controls for the phenotypes associated with familial longevity restricted to individuals with the APOE ε3/ε3 variant

	**Offspring N=921**	**Controls N=448**	**P-Value**
**Glucose-insulin homeostasis[a]**			
Glucose in mmol/L	5.7 ± 1.0	5.8 ± 1.1	**0.037**
Insulin in mU/L, median (IQR)	16.0 (9.0-28.0)	17.0 (9.0-28.0)	0.34
**Serum lipid levels[b]**			
Triglyceride in mmol/L, median (IQR)	*1.4 (1.0-2.1)*	1.5 (1.1-2.3)	**0.002**
HDL cholesterol in mmol/L	1.5 ± 0.4	1.4 ± 0.4	**0.016**
**Other serum levels**			
25-hydroxyvitamin D in nmol/L median (IQR)	63.9 (51.0-79.6)	68.3 (55.8-82.9)	**0.001**
**Disease History**			
Diabetes Mellitus, yes	35 (3.8)	36 (8.0)	**0.002**
Hypertension, yes	219 (23.8)	154 (34.4)	**<0.001**
Myocardial infarction, yes	18 (2.0)	15 (3.3)	0.15

Abbreviations: n, number of participants; IQR, interquartile range; SD, Standard deviation. Data presented as the mean ± SD unless indicated otherwise. [a]) Participants with diabetes were excluded. [b]) Participants using lipid lowering agents were excluded. Comparisons between offspring and partners were adjusted for age and gender, and corrected for familial relationships using clustered robust. P-values <0.05 are presented in bold.

Supplementary Table 3. Association (*APOE* variant-adjusted) residuals and LLS offspring/control status

	Model 1		Model 2	
	OR (95% CI)	P-Value	OR (95% CI)	P-Value
**Glucose-insulin homeostasis**[a]				
Glucose	0.88 (0.83 – 0.92)	**<0.001**	0.87 (0.83 – 0.92)	**<0.001**
Insulin	0.85 (0.76 – 0.95)	**0.003**	0.84 (0.76 – 0.94)	**0.003**
**Serum lipid levels**[b]				
Triglyceride	0.73 (0.62 – 0.87)	**<0.001**	0.75 (0.63 – 0.89)	**0.001**
HDL cholesterol	1.32 (1.05 – 1.67)	**0.017**	1.27 (1.01 – 1.61)	**0.042**
**Other serum levels**				
25-hydroxyvitamin D	0.56 (0.43 – 0.73)	**<0.001**	0.58 (0.44 – 0.75)	**<0.001**
